# A Randomized, Double-Blind, Controlled Phase III Clinical Trial to Evaluate the Immunogenicity and Safety of a Lyophilized Human Rabies Vaccine (Vero Cells) in Healthy Participants Aged 10–60 Years Following Essen and Zagreb Vaccination Procedures

**DOI:** 10.3390/vaccines11081311

**Published:** 2023-08-01

**Authors:** Xiaohong Wu, Jia Li, Lei Zhou, Jianmin Chen, Zhongqiang Jin, Qingwei Meng, Jing Chai, Hongxia Gao, Yunpeng Wang, Danhua Zhao, Heng Wu, Jieran Yu, Nan Chen, Yanan Wang, Yuan Lin, Peifang Huang, Yuhua Li, Yuhui Zhang

**Affiliations:** 1 Arbovirus Vaccine Department, National Institutes for Food and Drug Control, Beijing 102629, China; 2 School of Chemical Engineering, Dalian University of Technology, Dalian 116023, China; 3 General Manager Office, Dalian Aleph Biomedical Co., Ltd., Dalian 116620, China; 4 Vaccine Clinical Research Center of Sichuan Center for Disease Control and Prevention, Chengdu 610044, China; 5 Quality Control Department, Dalian Aleph Biomedical Co., Ltd., Dalian 116620, China; 6 Rabies Vaccine Production Workshop, Dalian Aleph Biomedical Co., Ltd., Dalian 116620, China; 7 Filling and Packaging Workshop, Dalian Aleph Biomedical Co., Ltd., Dalian 116620, China; 8 R&D Department, Dalian Aleph Biomedical Co., Ltd., Dalian 116620, China; 9 Department of Scientific Affairs, Simoon Record Beijing Co., Ltd., Beijing 100025, China; 10 Department of Project Management, Simoon Record Beijing Co., Ltd., Beijing 100025, China

**Keywords:** lyophilized human rabies vaccine (Vero cells), Essen and Zagreb procedures, immunogenicity, safety, non-inferiority

## Abstract

Objective: In this paper, we aim to show that the immunogenicity of the lyophilized human rabies vaccine (Vero cells) (investigational vaccine) developed by Dalian Aleph Biomedical Co., Ltd. in healthy participants aged 10–60 years old is non-inferior to the lyophilized PVRV (positive control) manufactured by Liaoning Chengda Biotechnology Co., Ltd. (Shenyang, China), and that its safety is clinically acceptable. Method: A total of 2776 participants were enrolled in this study and divided into four groups: a five-dose test group, a five-dose control group, a four-dose test group, and a four-dose control group. The patients in the four-dose groups (Zagreb) were vaccinated on Days 0 (two doses), 7 (one dose), and 21 (one dose), and those in the five-dose groups (Essen) were vaccinated on Days 0, 3, 7, 14, and 28 (one dose each). The rabies-virus-neutralizing antibody assay with the RFFIT was used to assess the immunogenicity, and the adverse events (AEs) and serious adverse events (SAEs) were identified and collated. Results: The positive seroconversion rate was up to 100% on Days 14 and 35/42 after vaccination following any procedures in pre-immunization antibody-negative participants, and the positive seroconversion rate and geometric mean concentration (GMC) of the test groups (Zagreb and Essen vaccination procedures) was not inferior to that of the control groups. On Day 7 after vaccination, the immunogenicity of the Zagreb procedure with two doses of the vaccine on Day 0 was superior to the Essen procedure with one dose of vaccine, that is, the former had a higher seroconversion rate and RVNA titer. The non-inferiority criterion of immunogenicity was met for the whole population, the population aged 10–18 years and ≥18 years, and the pre-immunization antibody-positive population. The incidences of all AEs, solicited AEs, and unsolicited AEs in both groups were not statistically significant, and no vaccination-related SAEs were observed. Conclusion: The investigated vaccine is safe, its immunogenicity is non-inferior to that of the control vaccine, and the efficacy of the Zagreb procedure is superior to that of the Essen procedure 7 days after the first dose.

## 1. Introduction

Rabies is a zoonosis caused by a rabies virus infection mainly spread when an infected animal bites or scratches a person or licks a person’s skin [1]. Rabies was discovered more than 4000 years ago, affects 150 countries and regions worldwide, and causes an estimated annual loss of USD 8.6 billion globally [2]. According to statistics, nearly 59,000 people worldwide die of rabies every year [3]. Nowadays, 95% of rabies cases worldwide occur in Asia and Africa, and almost all rabies victims are bitten by dogs [4,5,6]. China is a country that faces a severe rabies threat. In 2007, China reported 3300 cases of rabies [6]. In China, effective measures to control rabies include strengthening epidemic surveillance, issuing norms and guidelines for rabies exposure disposal, promoting the inclusion of the rabies vaccine and passive immunization preparations in medical insurance, and standardizing the behavior of urban dog breeding [6]. However, there are still no treatments for the clinical symptoms of rabies, and death is virtually the only endpoint for a patient developing any clinical symptoms of rabies [7].

Rabies differs from other infectious diseases in humans because timely and effective vaccination against rabies can prevent its onset, even after exposure to the virus. In China, with the increase in immunization with rabies vaccines, the incidence of rabies decreased from 0.047/100,000 people in 2016 to 0.014/100,000 people in 2020 [8]. Cell-based rabies vaccines, such as the purified Vero cell rabies vaccine (PVRV) approved in the mid-1980s, have been proven to be safe and effective for vaccinating millions of people worldwide for more than 40 years [9]. In addition, compared to the risks associated with the existing PVRVs, the biosafety risks related to the use of serum (contamination by bacteria, fungi, mycoplasma, and bovine viruses, as well as the induction of hypersensitivity) can be avoided during the manufacture of lyophilized PVRVs, which are expected to improve vaccination safety and reduce adverse side effects [10]. Lyophilized PVRVs have been successfully manufactured in China. For example, the lyophilized PVRVs manufactured by Liaoning Chengda Biotechnology Co., Ltd. (Shenyang, China) and Liaoning Yisheng Biopharm Co., Ltd. (Shenyang, China) have been approved by the National Health Commission of the People’s Republic of China and the former State Food and Drug Administration (SFDA, now the National Medical Products Administration (NMPA)) and have been sold and widely used throughout the country [11,12]. Studies have shown that both vaccines are effective at the WHO-recommended dose of ≥2.5 IU per single intramuscular injection (IM), and the concentration of the induced serum rabies virus neutralizing antibodies (RVNA) is ≥0.5 IU/mL.

Rabies vaccines can be classified into pre-exposure prophylaxis (PrEP) and post-exposure prophylaxis (PEP) [13]. As recommended by the WHO, there are two IM regimens for PEP: the five-dose regimen (Essen, 1-1-1-1-1) and the four-dose regimen (Zagreb, 2-1-1) [13,14]. According to studies on other rabies vaccines, both regimens have performed well in terms of safety and immunogenicity [15]. The Zagreb procedure has been widely used in clinical practice due to the lower number of injections required, lower cost, higher patient compliance, and earlier establishment of protection [16,17,18]. In this study, a randomized, double-blind, phase III clinical trial was conducted to evaluate the safety and immunogenicity of a lyophilized PVRV developed and manufactured by Dalian Aleph Biomedical Co., Ltd., and it was compared with the lyophilized PVRV from Liaoning Chengda Biotechnology Co., Ltd., which is widely used in China. This study aimed to show that the investigated vaccine had immunogenicity that is non-inferior to the control vaccine, has clinically acceptable safety, and that the early (Day 7) immunogenicity of the Zagreb procedure is superior to that of the Essen procedure. The goal of this clinical trial was to provide a basis for the implementation of the Zagreb procedure using the lyophilized vaccine.

## 2. Materials and Methods

### 2.1. Study Design

This study was designed to be a randomized, blinded, active control trial to evaluate the immunogenicity and safety of the lyophilized human PVRV developed by Dalian Aleph Biomedical Co., Ltd. in a population aged 10–60 years following a five-dose procedure and a four-dose procedure (China Clinical Trial ID: CTR20200042). The study was conducted from 4 August 2020 (date of enrollment of the first participant) to 17 September 2021 (date of the last visit of the last participant), with the data collected at the Sichuan Center for Disease Control and Prevention. No major changes to the test method were made after the start of the study: the endpoint indicators were not changed, and no interim analyses, interruptions, or discontinuations occurred. The contract research organization of this trial was Simoon Record Beijing Co., Ltd., and the testing institution for blood samples was the National Institutes for Food and Drug Control. The data management and statistical analyses were performed by Beijing Key Tech Statistical Consulting Co., Ltd. (Beijing, China).

### 2.2. Study Population

The eligibility criteria for participants were participants aged 10–60 years, with a legal identity certificate available, who voluntarily agreed to participate in the study and signed an informed consent form (participants or their guardians) and were able to understand the study procedures and take part in all planned visits. The main exclusion criteria were the following items: (1) participants who had a history of rabies vaccine immunization or use of passive immunization preparations against the rabies virus; (2) participants who were bitten (wounded skin) by animals susceptible to the rabies virus (such as dogs and cats) within 1 year before the first dose of the vaccine; (3) participants who had received any vaccines within 14 days before the first dose of the vaccine; (4) participants who had a history of severe allergy to any component of the investigational vaccine and any history of serious side effects caused by vaccines or drugs; (5) participants who were diagnosed with immunodeficiency or had received immunosuppressive therapy in the past 3 months; (6) participants who had received blood or blood-related products 3 months before enrollment; (7) participants who had a personal history or a family history of convulsions, epilepsy, encephalopathy, or psychosis; and (8) participants who had any conditions that might interfere with the assessment of the study objectives as considered by the investigator.

### 2.3. Randomization

In this study, 2776 participants were planned to be enrolled. A statistician first used SAS statistical software to randomize 2776 serial numbers via the stratified block randomization method. The stratified factor was the blood sampling scheme (blood sampling on days 0, 7, and 35/42, blood sampling on days 0, 14, and 35/42). The ratio of the 4-dose test group (T4), 4-dose control group (C4), 5-dose test group (T5), and 5-dose control group (C5) in each layer was 1:1:1:1. The vaccine samples were taken out from the original packaging and repackaged with a unified small box to achieve sample blinding. The statistician in charge of the random design and the relevant personnel loaded the research vaccine or control vaccine in each small box with a numbered label according to the random assignment table, and then transported these vaccines to each test site. The on-site researchers strictly assigned serial numbers to qualify the participants in the order of enrollment and then obtained and administered the corresponding vaccine sample based on the number.

### 2.4. Blinding

This trial was designed following the blinding method. The vaccine label was pasted at the designated position on the vaccine after being blinded by the statistician, who stored the blinded information and kept it confidential. The investigators and participants were blinded throughout the trial, and the randomization information of the trial was not accessible to the participants and/or their legal guardians, the investigators, or project members involved in any endpoint evaluation, data review, or data analysis of the study. The vaccinations were performed by investigators who were blinded.

### 2.5. Investigational Vaccine

The investigational vaccine was the Lyophilized Human Rabies Vaccine (Vero cells) manufactured by Dalian Aleph Biomedical Co., Ltd., which was prepared using Vero cells cultured on a sheet carrier by inoculating the fixed rabies virus CTN-1V strain (fixed strain) in a bioreactor following culture, harvest, concentration, virus inactivation, purification, and then lyophilization. Its active ingredient was inactivated rabies virus (CTN-1V strain), and its excipients were sodium chloride, potassium chloride, potassium dihydrogen phosphate, disodium hydrogen phosphate, sucrose, and human blood albumin. After reconstitution, each vial contained 0.5 mL of the vaccine, and the batch number was DG201908001. The control vaccine was the Lyophilized Human Rabies Vaccine (Vero cells) manufactured by Liaoning Chengda Biotechnology Co., Ltd., which was prepared using Vero cells cultured on a sheet carrier by inoculating the fixed rabies virus L. Pasteur PV2061 strain (fixed strain) in a bioreactor following culture, harvest, concentration, virus inactivation, purification, and then lyophilization. Its active ingredient was inactivated rabies virus, and its excipients were sodium chloride, disodium hydrogen phosphate, sodium dihydrogen phosphate, human blood albumin, and Dextran 40. The batch number was 201906167, and the strength and active ingredient were consistent with those of the investigational vaccine. After being synchronously detected by Dalian Aleph Biomedical Co., Ltd., the potencies of the investigational vaccine and the control vaccine were 5.2 IU/dose and 5.0 IU/dose, respectively.

### 2.6. Sample Size

In this study, a step-down strategy was used to sequentially evaluate the non-inferiority of the antibody-positive seroconversion rates and antibody geometric mean concentration (GMC) on Day 14 after the first dose of vaccination in T5 and C5 as well as in T4 and C4. With a test level of one-sided α = 0.025 and the power controlled at 85%, it was found that 295 cases were needed in each of the 4 groups, with a total of 1180 cases required. Considering that half of the participants were sampled on Day 7/14 after the first dose and the dropout rate was about 15%, the planned sample size for each group in the second period was [295/(1 − 0.15)] × 2 = 694 cases (“2” represents two blood-sampling schemes), with a total of 2776 cases planned for the 4 groups.

### 2.7. Study Population, Grouping, and Vaccination

A total of 2776 participants were enrolled in the study, with 694 cases each in T4 and C4, and they were administered two doses of the vaccine (test vaccine or control vaccine) on Day 0 and one dose each on Days 7 and 21. In addition, 694 participants were included in T5 and C5, who were administered one dose of the vaccine (test vaccine or control vaccine) each on Days 0, 3, 7, 14, and 28.

### 2.8. Study Endpoints

#### 2.8.1. Primary Endpoints

Immunogenicity Endpoint: The positive seroconversion rates of antibodies and the GMC of antibodies in each group 14 days after the first vaccination dose.

Safety Endpoints: The incidence of solicited adverse events (AEs), including vaccination site (local) AEs and non-vaccination site (systemic) AEs, within 0–7 days after each dose; the incidence of unsolicited AEs within 0–30 days after each dose; and the incidence of all serious adverse events (SAEs) from the first dose to 6 months after the full course of immunization.

#### 2.8.2. Secondary Endpoints

The secondary endpoints were the positive seroconversion rates of antibodies and the GMC of antibodies for all the participants 7 days after the first dose of vaccination and the positive seroconversion rates of antibodies and the GMC of antibodies for all the participants 14 days after the full course of immunization.

Here, the positive seroconversion rate of antibodies was defined as the percentage of participants with rabies-virus-neutralizing antibody (RVNA) before immunization < 0.5 IU/mL showing RVNA ≥ 0.5 IU/mL after immunization. The pre-immunization antibody-positive value was defined as RVNA ≥ 0.5 IU/mL before the first vaccination dose. The antibody-negative value was defined as rabies-virus-neutralizing antibody < 0.5 IU/mL before the first vaccination dose.

### 2.9. Statistical Analysis

The immunogenicity analysis was performed according to the per-protocol set (PPS) in the subgroups by age group, pre-immunization antibody-positive or antibody-negative populations, and populations aged 10–18 years or ≥18 years, without correction analyses. The Clopper–Pearson method was used to calculate the bilateral 95% CI of the positive seroconversion rate of antibodies, and the difference between groups was statistically tested using the Chi-square test/Fisher exact probability method. For the non-inferiority cut-off value of the positive seroconversion rate of antibodies, the lower 95% CI for the difference in the positive seroconversion rate of the neutralizing antibody (test group–control group) should be greater than −5%. For the superiority cut-off value of the positive seroconversion rate of antibodies, the lower 95% CI for the difference in the positive seroconversion rate of the neutralizing antibody (T4–T5) should be greater than 0. The two-sided 95% CIs of the antibody GMC, GMC fold change, and GMC ratio (test group/control group) after logarithmic transformation were statistically tested using analysis of covariance (ANCOVA) and paired t-tests. For the non-inferiority cut-off value of antibody GMC, the lower 95% CI of the neutralizing antibody GMC ratio (test group/control group) should be greater than 0.67.

Safety analysis was performed using the safety set (SS). AEs and SAEs were medically coded using MedDRA (Version: 24.1, International Council for Harmonisation of Technical Requirements for Pharmaceuticals for Human Use, ICH). The solicited AEs were statistically summarized as systemic AEs and local AEs. The cases and incidences of all AEs and SAEs in each group were calculated. Fisher’s exact test was used to identify the differences between the two dosing groups.

The statistical software SAS Version 9.4 (SAS Institute Inc., Cary, NC, USA) was used for all statistical analyses. The difference was considered statistically significant at *p* < 0.05.

### 2.10. Serological Methods

All serum samples were collected by the National Institutes for Food and Drug Control in strict accordance with regulations and laboratory manuals using the rapid fluorescent focus inhibition test (RFFIT) to detect rabies-virus-neutralizing antibodies.

RFFIT is the standard method recommended by the WHO for the detection of rabies-virus-neutralizing antibodies (RVNAs). The rabies virus CVS strain was used as the challenge virus neutralizing the RVNAs in the serum samples. A suspension of susceptible cells containing viral residues was used to detect the viruses by fluorescent antibody staining. A fixed amount of CVS viruses was incubated with continuously diluted serum samples (neutralization in vitro), which were then added to the suspension of susceptible cells. After incubation for 24 h, the cells were fixed with a monolayer of acetone and stained with fluorescently labeled anti-NP antibodies to detect unneutralized viruses. The dilution required to reduce the concentration of the fixed amount of CVS viruses (FFU/mL) by 50% was calculated by comparing it with the viral control group, and the potency of neutralizing antibodies in each serum sample was determined through comparison with the reference serum with a known potency.

## 3. Results

### 3.1. Study Population

A total of 2776 participants were included in the trial, of whom 2671 participants completed the trial and 105 cases dropped out: 2 cases due to SAEs, 11 cases due to non-SAEs, 6 cases due to protocol violations, 61 cases due to voluntary withdrawal without any AEs, 14 cases because they left the region of the study site, 3 cases due to loss of follow-up, and 8 cases due to other reasons (Table 1, Appendix A, Table A1 and Table A2). The FAS set included 694 participants from T5, 691 participants from C5, 688 participants from T4, and 691 participants from C4, and no changes were made to the groupings. In addition, the ages, genders, heights, and weights of the participants in each group were evenly distributed among all of the participants and between the age groups (Table 2 and Appendix A, Table A3).

### 3.2. Immunogenicity

**Figure 1:**
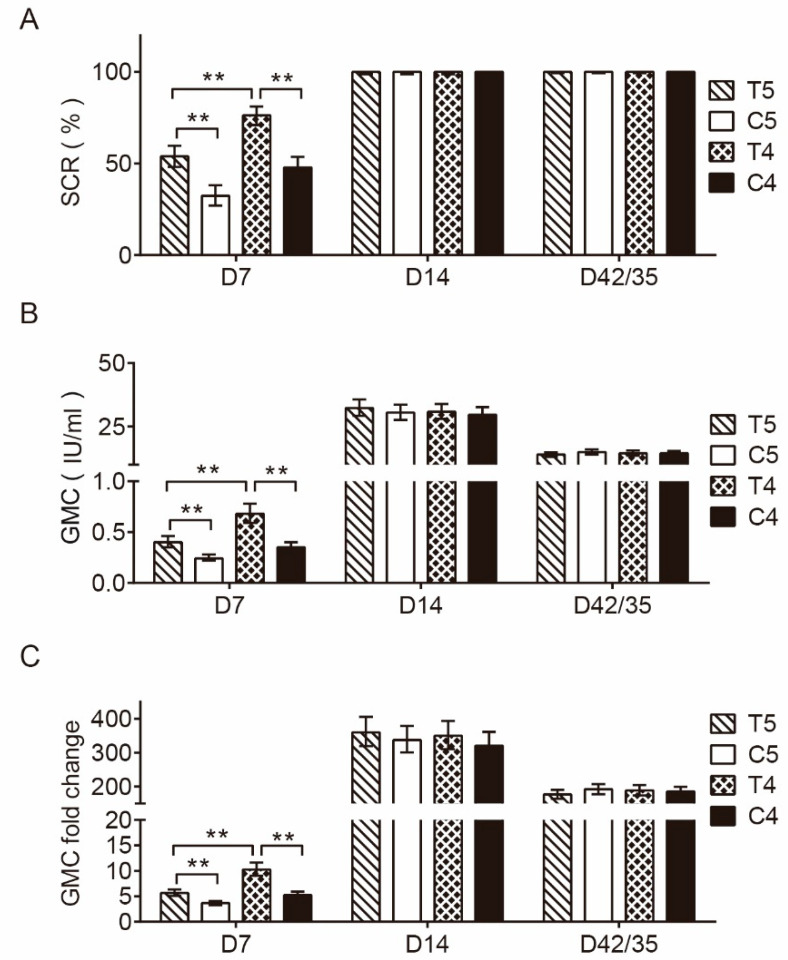
Neutralizing antibody SCR, GMC, and GMC fold change after immunization in the pre-immunization antibody-negative population. (**A**) Positive seroconversion rates of neutralizing antibodies in participants of each group. SCR = seroconversion rates—that is, the percentage of the number of participants with rabies-virus-neutralizing antibody (RVNA) ≥ 0.5 IU/mL after immunization among the total number of participants with RVNA < 0.5 IU/mL before immunization; or the percentage of participants with an RVNA titer that increased at least 4 times after immunization among the total number of participants with RVNA ≥ 0.5 IU/mL before immunization. **, *p* < 0.001. (**B**) GMC levels of neutralizing antibodies in participants of each group. GMC: geometric mean concentration. **, *p* < 0.001. (**C**) Neutralizing antibody GMC fold change in participants of each group. **, *p* < 0.001.

Figure 1A,B presents the positive seroconversion rates of neutralizing antibodies after immunization in the pre-immunization antibody-negative population and the GMC levels, respectively. The results show that the positive seroconversion rates and the GMC levels in T5 were higher than those in C5, and the positive seroconversion rates and the GMC levels in T4 were higher than those in C4 and T5 at 7 days after the first dose of immunization (PPS1); the RVNA ranges in T5, C5, T4, and C4 were (<0.1, 80.8), (<0.1, 72.4), (<0.1, 197.4), and (<0.1, 76.2), respectively.

The positive seroconversion rates of neutralizing antibodies in T5, C5, T4, and C4 were 100.00% each. At 14 days after the first dose of immunization (PPS2) and at 14 days after the full course of immunization (PPS3), the differences in the positive seroconversion rates between T5 and C5 were 0.00% (95% CI: −1.30%, 1.28%) and 0.00% (95% CI: −0.63%, 0.66%), respectively, the differences between T4 and C4 were 0.00% (95% CI: −1.29%, 1.32%) and 0.00% (95% CI: −0.66%, 0.64%), respectively, and the differences between T4 and T5 were 0.00% (95% CI: −1.29%, 1.30%) and 0.00% (95% CI: −0.66%, 0.63%). The differences showed that the lower limits of all the 95% CIs of the positive seroconversion rates were greater than −5% (Figure 1A).

In addition, at 14 days after the first dose of vaccination (PPS2), the neutralizing antibody GMC levels in the serum were 32.30 IU/mL (95% CI: 29.25 IU/mL, 35.68 IU/mL) and 30.84 IU/mL (95% CI: 28.08 IU/mL, 33.88 IU/mL) in T5 and T4, respectively, increasing by 359.84 times and 320.46 times compared with those before vaccination, respectively; the RVNA ranges in T5, C5, T4, and C4 were (1.7, 1231.5), (2.6, 1231.5), (3.1, 326.4), and (1.5, 493), respectively. At 14 days after the full course of immunization (PPS3), the GMC levels were 13.97 IU/mL (95% CI: 13.09 IU/mL, 14.91 IU/mL) and 14.54 IU/mL (95% CI: 13.56 IU/mL, 15.58 IU/mL), increasing by 176.51 and 188.73 times compared with those before immunization, respectively (Figure 1B,C); and the RVNA ranges in T5, C5, T4, and C4 were (1.2, 493), (1.7, 333), (1.7, 999), and (0.9, 410.5), respectively.

Furthermore, at 14 days after the first dose of vaccination (PPS2) and 14 days after the full course of immunization (PPS3), the GMC ratios between T5 and C5 were 1.06 (95% CI: 0.92, 1.22) and 0.93 (95% CI: 0.85, 1.02), respectively, the GMC ratios between T4 and C4 were 1.04 (95% CI: 0.91, 1.19) and 1.00 (95% CI: 0.91, 1.10), respectively, and the GMC ratios between T4 and T5 were 0.95 (95% CI: 0.83, 1.09) and 1.04 (95% CI: 0.95, 1.14), showing that all the lower 95% CIs of the GMC ratios were greater than 0.67.

In terms of the total population, the population aged 10–18 years old, the population ≥ 18 years, and the pre-immunization antibody-negative population, the lower 95% CIs of the differences in the positive seroconversion rate between the five-dose groups, between the four-dose groups, and between T4 and T5 were all greater than −5%, and the lower 95% CIs of the GMC ratio were all greater than 0.67 (Appendix A, Table A4 and Table A5).

### 3.3. Safety

Table 3 presents the incidences of AEs in each group. The results show that the incidences of AEs were 36.80%, 35.41%, 37.52, and 34.34% and the incidences of AEs associated with vaccination were 33.77%, 31.35%, 33.19%, and 29.44% in T4, C4, T5, and C5, respectively. In addition, in T4, the incidence of systemic AEs was 11.40%, mainly characterized by headache (4.33%), and the incidence of local AEs was 28.72%, mainly characterized by pain at the vaccination site (27.13%). In T5, the incidence of systemic AEs was 12.41%, mainly characterized by vertigo (3.90%), and the incidence of local AEs was 28.14%, mainly characterized by pain at the vaccination site (26.98%). There was no statistical difference in the above-mentioned AEs between groups, with the exception that the incidences of local AEs and pain in T5 were slightly higher than those in C5. In addition, no vaccine-related SAEs were observed.

## 4. Discussion

In this study, the immunogenicity and safety of the lyophilized PVRV developed by Dalian Aleph Biomedical Co., Ltd. in a population aged 10–60 years following the Essen and Zagreb procedures were evaluated by comparing them with those of the lyophilized PVRV manufactured by Liaoning Chengda Biotechnology Co., Ltd. This study showed that the investigational vaccine was non-inferior to the positive control vaccine in both the Essen procedure and the Zagreb procedure. Moreover, the efficacy of the investigational vaccine administered according to the Zagreb procedure was superior to that of the Essen procedure shortly (Day 7) after vaccination, and the investigational vaccine showed good safety in both procedures.

Regarding immunogenicity, the alternative endpoints and evaluation criteria play an important role in evaluating rabies vaccines based on the RVNA-positive seroconversion rate and the GMC. In this study, as per the immunogenicity analysis of the pre-immunization antibody-negative population, the positive seroconversion rate of antibodies was 100.00% in both the five-dose group and the four-dose group 14 days after the first dose of vaccination and 14 days after the full course of immunization, which was consistent with the result that protective antibodies were rapidly generated following five-dose rabies vaccine immunization in a previous study [19], showing the reliability of this study in terms of the positive seroconversion rate of lyophilized PVRVs. In addition, the GMC in the participants gradually increased after vaccination and peaked on Day 14 (both greater than 28.08 IU/mL), much greater than the WHO specification for the effective protection capacity of rabies vaccines (RVNA ≥ 0.5 IU/mL). On Day 7 after vaccination, the GMC of the T4 group exceeded 0.68 IU/mL, which was higher than 0.40 IU/mL of T5 in this study. In combination with the similar GMC data in this study between the four-dose group and the five-dose group 14 days after the first dose of vaccination and the full course of immunization, we believe that the four-dose procedure in the early stage of immunization was superior to the five-dose procedure, which is consistent with the conclusion reported by Shen et al. [20]. Meanwhile, in the subgroup analysis between the populations aged 10–18 years and ≥18 years, it was found that the populations belonging to both age groups had high positive seroconversion rates of antibodies and GMC levels of antibodies after being administered the investigational vaccine, which was similar to the findings by Li et al. [21]. However, the positive rates and the GMC levels were higher in the population aged 10–18 years and ≥18 years old, different from the study by Fang et al. [22]. All these results indicate that the investigational lyophilized PVRV in this study had good immunogenicity that was not inferior to the control vaccine.

In terms of safety analysis, the incidence of AEs in each test group was similar to that in the control group, and the incidence of AEs in T5 was 37.52%, which was similar to the 36.8% reported by Shen et al. [20]. In addition, there was no significant difference in the incidences of local and systemic reactions among the groups. In this study, pain at the injection site was the most common local symptom, while headache was the most common systemic symptom, consistent with the studies by Wang et al. [23], Zhang et al. [15], and Pichon et al. [24]. Another common systemic adverse reaction was vertigo, of which the recovery was within 2 weeks in all cases. In addition, no vaccine-related SAEs were reported in this study, especially those observed in previous case reports, such as severe allergic reactions [22] and acute disseminated encephalomyelitis [25], indicating the good safety of the investigational vaccine.

In this comparative clinical trial involving both the Essen and Zagreb vaccination procedures and a control vaccine, participants of different age groups and the pre-immunization antibody-negative and -positive conditions were fully investigated, and multiple factors affecting the vaccination results were excluded. This clinical trial is a simulated exposure study in healthy participants, and the vaccine’s efficacy in the post-exposure population may be monitored after the vaccine is marketed.

## 5. Conclusions

This study has demonstrated that the lyophilized PVRV manufactured by Dalian Aleph Biomedical Co., Ltd. is not inferior to the lyophilized PVRV manufactured by Liaoning Chengda Biotechnology Co., Ltd. in terms of immunogenicity, with safety similar to that of the vaccine. In addition, the groups undergoing the Zagreb vaccination procedure showed higher GMC levels 7 days after the first vaccination dose than those undergoing the Essen vaccination procedure. In short, the results of this study indicate that the investigational vaccine administered following the Essen and Zagreb vaccination procedures is safe, with good immunogenicity, and the Zagreb vaccination procedure produces protection earlier than the Essen vaccination procedure, providing a valuable alternative for the prevention of rabies.

## Figures and Tables

**Table 1:** Enrollment, completion of the trial, and inclusion in each statistical analysis dataset.

Analysis Item	T_5_	C_5_	T_4_	C_4_
Screened	3128			
Randomized	694	694	694	694
Vaccination				
First dose	693	693	693	689
Second dose	674	674	693	689
Third dose	672	670	678	674
Fourth dose	672	666	674	673
Fifth dose	668	661	/	/
Blood sampling				
Pre-immunization ^1^	694	691	688	691
7 days after vaccination of the first dose ^2^	333	325	332	332
14 days after vaccination of the first dose ^2^	334	337	343	342
14 days after the full vaccination ^1^	669	661	664	672
Dropout	25	35	23	20
SAEs	0	2	0	0
Non-SAEs	3	5	1	2
Protocol violation	0	1	3	2
Voluntary withdrawal without AEs	12	22	13	12
Leaving the region of the study site	6	2	4	2
Loss to follow-up	3	0	0	0
Other	1	3	2	2
Completion of the trial	668	659	671	673
Number of participants included in each analysis dataset				
SS	693	693	693	689
FAS	694	691	668	691
PPS1	324	316	321	323
PPS2	329	329	333	331
PPS3	659	643	653	663

Note: ^1^: Blood sampling from all participants; ^2^: blood sampling from 50% of participants before the third dose and from the other 50% of participants before the fourth dose. FAS: full analysis set; SS: safety set; PPS1: per-protocol set 1, which includes all the participants who were administered the first two doses of the vaccine, completed immunogenicity blood sampling prior to vaccination and 7 days after the first dose, and had effective antibody testing values; PPS2: per-protocol set 2, which includes all the participants who were administered the first three doses of the vaccine, completed immunogenicity blood sampling prior to vaccination and 14 days after the first dose, and had effective antibody testing values; PPS3: per-protocol set 3, which includes all the participants who had completed the full course of immunization and immunogenicity blood sampling prior to vaccination and 14 days after the full course of immunization and had effective antibody testing values.

**Table 2:** Demographic data and baseline clinical characteristics (FAS) of the study participants.

Analysis Item	T5 (N = 694)	C5 (N = 691)	T4 (N = 688)	C4 (N = 691)	*p* Value
Age (year)					
Mean (SD)	43.1 (12.3)	43.0 (12.3)	42.8 (12.7)	43.4 (12.2)	0.8857
Gender					
Female, n (%)	470 (67.72)	465 (67.29)	472 (68.60)	473 (68.45)	
Height (cm)					
Mean (SD)	156.1 (7.8)	156.4 (8.2)	156.0 (8.2)	155.9 (7.9)	0.6202
Weight (kg)					
Mean (SD)	60.51 (10.82)	60.12 (10.95)	59.90 (10.95)	59.74 (11.23)	0.5819

**Table 3:** Incidences of adverse events in all participants.

Adverse Event	T4 (N = 693)	C4 (N = 689)	T5 (N = 693)	C5 (N = 693)	* P1	* P2	* P3
All adverse events	36.80	35.41	37.52	34.34	0.2397	0.6144	0.8241
Adverse events related to the investigational vaccine	33.77	31.35	33.19	29.44	0.1476	0.3585	0.8644
Solicited AEs	33.48	31.06	32.76	29.29	0.1817	0.3573	0.8194
Non-vaccination-site (systemic) AEs	11.40	11.32	12.41	13.13	0.7476	1.0000	0.6188
Fever	2.74	2.76	0.14	2.45	0.8625	1.0000	1.0000
Asthenia	2.02	3.48	2.16	4.33	0.0618	0.1024	1.0000
Headache	4.33	3.63	3.32	4.18	0.4717	0.5825	0.4009
Nausea	1.30	0.87	1.44	2.60	0.2431	0.6053	1.0000
Vomiting	0.43	0.00	0.43	0.87	0.7258	0.2495	1.0000
Vertigo	2.60	3.05	3.90	4.62	0.6877	0.6301	0.2250
Abdominal pain	0.58	1.16	0.43	1.30	0.1442	0.2635	1.0000
Arthralgia	1.44	1.45	2.02	1.30	0.4009	1.0000	0.5377
Muscle pain	2.45	2.47	2.16	2.16	1.0000	1.0000	0.8584
Acute allergic reaction	0.14	0.44	0.72	0.14	0.3741	0.3730	0.2177
Vaccination-site (local) AEs	28.72	26.71	28.14	23.38	0.0493	0.4347	0.8582
Pain	27.13	25.40	26.98	22.08	0.0393	0.5014	1.0000
Induration	0.00	0.29	0.00	0.58	0.1245	0.2484	1.0000
Redness	0.72	0.00	0.87	0.72	1.0000	0.7258	1.0000
Swelling	1.30	0.87	1.44	0.87	0.4518	0.6053	1.0000
Pruritus	3.61	3.63	3.46	4.04	0.6719	1.0000	1.0000
Rash	0.00	0.15	0.00	0.14	1.0000	0.4986	1.0000
Unsolicited AEs	0.58	0.58	0.72	0.87	1.0000	1.0000	1.0000
AEs not related to the investigational vaccine	7.07	6.53	8.51	9.52	0.5738	0.7488	0.3672
Grade 3 or higher AEs	0.72	0.87	0.58	1.44	0.1774	0.7729	1.0000

Note: * P1: comparison between T5 and C5; P2: comparison between T4 and C4; P3: comparison between T4 and T5.

## Data Availability

The data presented in this study are available from the corresponding author upon request. The data are not publicly available due to privacy and ethical considerations.

## Appendix A

**Table A1:** SAEs leading to dropouts.

SAE Description	System Organ Class	Preferred Term	Start Date	End Date	Duration	Days from Vaccination	Correlation	SAE Leading to	Outcome	Leading to Dropouts?
Participant Serial Number: 0733; Age Group: 18–60-year-old group; Group: C5; Age: 53 years; Gender: male; First Dose Date: 13 November 2020										
Acute brain stem infarction: unconsciousness, weakness in the right limb, distortion of commissure, slurred speech, and gatism. Head CT: cavitary hard foci in the right cerebellar hemisphere; a few cavitary hard foci in the bilateral basal ganglia and right cerebellar hemisphere, and bone density shadow of nodule in the left ethmoid sinus; foci of brain stem infarction and inflammation in the bilateral ethmoid sinuses; foci of brain stem infarction; and inflammatory changes in the bilateral ethmoid sinuses and sphenoid sinuses.	Nervous system disorders	Brain stem infarction	16 February 2021	21 March 2021	34	96	Not related	Hospitalization or prolonged hospitalization	Death	Yes
Participant Serial Number: 2349; Age Group: 18–60-year-old group; Group: C5; Age: 52 years; Gender: female; First Dose Date: 18 November 2020										
Gallstones with chronic cholecystitis: sudden upper right quadrant pain for 1+ days, pain and discomfort in the upper abdomen, persistent dull pain, paroxysmal exacerbation, nausea and retching, obvious tenderness in the upper right quadrant, slight rebound tenderness, slight percussion pain in the liver region, coarse hepatic parenchymal echoes, cholecystitis, gallstones, gallbladder enlargement and effusion, gastrointestinal pneumatosis, neutrophil ratio 89.2% ↑, total bilirubin 28.75 umol/L ↑ * , direct bilirubin 15.87 umol/L ↑, amylase 225.80 U/L ↑, glutamic–pyruvate transaminase 173.10 U/L ↑, glutamic–oxaloacetic transferase 163 U/L ↑, glutamyltransferase 113.40 U/L ↑, alkaline phosphatase 135.5 U/L ↑, and amylase in urine 616.60 U/L ↑.	Hepatobiliary disorders	Cholecystitis, chronic	25 November 2020	4 December 2020	10	8	Unlikely	Hospitalization or prolonged hospitalization	Recovered	Yes
Gallstones with chronic cholecystitis: sudden upper right quadrant pain for 1+ days, pain and discomfort in the upper abdomen, persistent dull pain, paroxysmal exacerbation, nausea and retching, obvious tenderness in the upper right quadrant, slight rebound tenderness, slight percussion pain in the liver region, coarse hepatic parenchymal echoes, cholecystitis, gallstones, gallbladder enlargement and effusion, gastrointestinal pneumatosis, neutrophil ratio 89.2% ↑, total bilirubin 28.75 umol/L ↑, direct bilirubin 15.87 umol/L ↑, amylase 225.80 U/L ↑, glutamic–pyruvate transaminase 173.10 U/L ↑, glutamic–oxaloacetic transferase 163 U/L ↑, glutamyltransferase 113.40 U/L ↑, alkaline phosphatase 135.5 U/L ↑, and amylase in urine 616.60 U/L ↑.	Hepatobiliary disorders	Cholelithiasis	25 November 2020	4 December 2020	10	8	Unlikely	Hospitalization or prolonged hospitalization	Recovered	Yes

Note: * The upward arrow “↑” indicates an increase.

**Table A2:** Non-SAEs leading to dropouts.

Adverse Event Description	System Organ Classes	Preferred Term	Types of Adverse Events	Number of Vaccinations	Start Date	End Date	Date of Vaccination	Duration Days	Days from Vaccination	Is it within 30 min?	Severity	Treatment Situation	Correlation
Participant Serial Number: 0147, Age Group: 18–60 years, Group: C4, Age: 40 years, Gender: Male													
Pain	Systemic diseases and various reactions at the administration site	Pain at the inoculation site	Solicited //Adverse events at the inoculation site (local)	1/2	23 October 2020	27 October 2020	23 October 2020	5	0	No	Grade 2	Without medication	May be related
Pain	Systemic diseases and various reactions at the administration site	Pain at the inoculation site	Solicited //Adverse events at the inoculation site (local)	1/2	23 October 2020	27 October 2020	23 October 2020	5	0	No	Grade 2	Without medication	May be related
Participant Serial Number: 0185, Age Group: 18–60 years, Group: C5, Age: 47 years, Gender: Female													
Vomiting	Gastrointestinal system diseases	Vomiting	Solicited //Non-inoculation site (systemic) adverse events	4	6 November 2020	6 November 2020	6 November 2020	1	0	No	Grade 2	Outpatient medication treatment	May be related
Nausea	Gastrointestinal system diseases	Nausea	Solicited //Non-inoculation site (systemic) adverse events	4	6 November 2020	6 November 2020	6 November 2020	1	0	No	Grade 2	Outpatient medication treatment	May be related
Pain	Systemic diseases and various reactions at the administration site	Pain at the inoculation site	Solicited //Adverse events at the inoculation site (local)	4	6 November 2020	9 November 2020	6 November 2020	4	0	No	Grade 1	Self-medication treatment	May be related
Rash	Systemic diseases and various reactions at the administration site	Inoculation site rash	Solicited //Adverse events at the inoculation site (local)	4	6 November 2020	11 November 2020	6 November 2020	6	0	No	Grade 3	Self-medication treatment	May be related
Indurate	Systemic diseases and various reactions at the administration site	Inoculation site hardening	Solicited //Adverse events at the inoculation site (local)	4	6 November 2020	9 November 2020	6 November 2020	4	0	No	Grade 2	Self-medication treatment	May be related
Flush	Systemic diseases and various reactions at the administration site	Vaccination site erythema	Solicited //Adverse events at the inoculation site (local)	4	6 November 2020	9 November 2020	6 November 2020	4	0	No	Grade 3	Self-medication treatment	May be related
Swelling	Systemic diseases and various reactions at the administration site	Swelling of the vaccination site	Solicited //Adverse events at the inoculation site (local)	4	6 November 2020	11 November 2020	6 November 2020	6	0	No	Grade 3	Self-medication treatment	May be related
Abdominal pain	Gastrointestinal system diseases	Abdominal pain	Solicited //Non-inoculation site (systemic) adverse events	4	6 November 2020	6 November 2020	6 November 2020	1	0	No	Grade 2	Outpatient medication treatment	May be related
Acute gastroenteritis	Infection and infectious diseases	Gastroenteritis	Unsolicited	4	6 November 2020	7 November 2020	6 November 2020	2	0	No	Grade 3	Outpatient medication treatment	May be unrelated
Participant Serial Number: 0199, Age Group: 18–60 years, Group: C5, Age: 50 years, Gender: Male													
Pain	Systemic diseases and various reactions at the administration site	Pain at the inoculation site	Solicited //Adverse events at the inoculation site (local)	2	26 October 2020	31 October 2020	26 October 2020	6	0	No	Grade 2	Without medication	May be related
Participant Serial Number: 1999, Age Group: 18–60 years, Group: C4, Age: 43 years, Gender: Female													
Erythema	Skin and subcutaneous tissue diseases	Contact dermatitis	Unsolicited	1/2	14 December 2020	19 December 2020	8 December 2020	6	6	No	Grade 2	Without medication	May be unrelated
Participant Serial Number: 2108, Age Group: 18–60 years, Group: T5, Age: 46 years, Gender: Female													
Breast nodules	Reproductive system and breast diseases	Breast lump	Unsolicited	1	2 November 2020	15 July 2021	31 October 2020	256	2	No	Grade 2	Outpatient medication treatment	May be unrelated
Breast tenderness	Reproductive system and breast diseases	Breast pain	Unsolicited	1	2 November 2020	5 January 2021	31 October 2020	75	2	No	Grade 2	Outpatient medication treatment	May be unrelated
Participant Serial Number: 2138, Age Group: 18–60 years, Group: T5, Age: 36 years, Gender: Female													
Pain	Systemic diseases and various reactions at the administration site	Pain at the inoculation site	Solicited //Adverse events at the inoculation site (local)	1	2 November 2020	6 November 2021	31 October 2020	5	2	No	Grade 1	Without medication	May be related
Participant Serial Number: 2188, Age Group: 18–60 years, Group: C5, Age: 44 years, Gender: Female													
Fever: 39.3 °C	Systemic diseases and various reactions at the administration site	Fever	Solicited //Non-inoculation site (systemic) adverse events	4	15 November 2020	17 November 2020	15 November 2020	3	0	No	Grade 3	Outpatient medication treatment	May be related
Headache	Various nervous system disease	Headache	Solicited //Non-inoculation site (systemic) adverse events	4	15 November 2020	15 November 2020	15 November 2020	1	0	No	Grade 3	Outpatient medication treatment	May be related
Nausea	Gastrointestinal system diseases	Nausea	Solicited //Non-inoculation site (systemic) adverse events	4	15 November 2020	15 November 2020	15 November 2020	1	0	No	Grade 1	Outpatient medication treatment	May be related
Participant Serial Number: 2265, Age Group: 18–60 years, Group: C5, Age: 51 years, Gender: Female													
Acute gastroenteritis	Infection and infectious diseases	Gastroenteritis	Unsolicited	1	2 November 2020	3 November 2020	1 November 2020	2	1	No	Grade 3	Outpatient medication treatment	May be unrelated
Participant Serial Number: 2405, Age Group: 18–60 years, Group: T5, Age: 48 years, Gender: Female													
Acute allergic reaction Other: rash with itching, redness, and swelling	Immune system diseases	Hypersensitivity	Solicited //Non-inoculation site (systemic) adverse events	4	6 December 2020	13 December 2020	5 December 2020	8	1	No	Grade 2	Outpatient medication treatment	May be related
Participant Serial Number: 2467, Age Group: 18–60 years, Group: C5, Age: 54 years, Gender: Male													
Asthenia	Systemic diseases and various reactions at the administration site	Asthenia	Solicited //Non-inoculation site (systemic) adverse events	3	11 December 2020	20 December 2020	8 December 2020	10	3	No	Grade 2	Without medication	May be related
Participant Serial Number: 2540, Age Group: 18–60 years, Group: T4, Age: 52 years, Gender: Female													
Pain	Systemic diseases and various reactions at the administration site	Pain at the inoculation site	Solicited //Adverse events at the inoculation site (local)	1/2	1 December 2020	4 December 2020	1 December 2020	4	0	No	Grade 1	Without medication	May be related
Pain	Systemic diseases and various reactions at the administration site	Pain at the inoculation site	Solicited //Adverse events at the inoculation site (local)	1/2	1 December 2020	4 December 2020	1 December 2020	4	0	No	Grade 1	Without medication	May be related

**Table A3:** Demographic data and baseline clinical characteristics (FAS) of each age group.

Analysis Item	Participants Aged 10–17 Years					Participants Aged 18–60 Years				
	T5 (N = 47)	C5 (N = 53)	C4 (N = 47)	T4 (N = 53)	*p*-Value	T5 (N = 47)	C5 (N = 53)	C4 (N = 47)	T4 (N = 53)	*p*-Value
Age (years)										
Mean (SD)	12.1 (1.4)	12.7 (2.0)	12.0 (1.9)	12.5 (2.0)	0.1964	45.3 (9.3)	45.5 (8.9)	45.4 (9.5)	45.7 (9.1)	0.9087
Gender										
Female, n (%)	22 (46.81)	27 (50.94)	22 (46.81)	24 (45.28)		448 (69.24)	438 (68.65)	448 (70.55)	451 (70.03)	
Height (cm)										
Mean (SD)	153.4 (10.1)	152.4 (11.0)	148.4 (11.4)	151.8 (12.5)	0.1615	156.3 (7.6)	156.8 (7.9)	156.3 (7.6)	156.4 (7.3)	0.7025
Weight (kg)										
Mean (SD)	49.52 (14.22)	44.86 (8.91)	43.13 (12.32)	45.58 (12.06)	0.0678	61.31 (10.09)	61.39 (10.13)	61.09 (9.97)	60.95 (10.14)	0.8540

**Table A4:** Comparison of positive seroconversion (four-fold growth) rates of neutralizing antibodies in the different subgroup populations.

Population	Blood Sampling Time (Analysis Set)	T5		C5		T4		C4 (95% CI)		Between T5s		Between T4s		Between T4 and T5s	
		N	Positive Seroconversion (Four-Fold Growth) Rate (95% CI)	N	Positive Seroconversion (Four-Fold Growth) Rate (95% CI)	N	Positive Seroconversion (Four-Fold Growth) Rate (95% CI)	N	Positive Seroconversion (Four-Fold Growth) Rate (95% CI)	Ratio Difference (95% CI)	*p*	Ratio Difference (95% CI)	*p*	Ratio Difference (95% CI)	*p*
Total population	7 days after the first dose of vaccine (PPS1)	324	53.70 (48.11, 59.23)	316	32.59 (27.45, 38.07	321	74.14 (68.99, 78.85)	323	49.23 (43.65, 54.82	21.11 (13.50, 28.47)	<0.0001	24.92 (17.54, 32.03)	<0.0001	41.55 (34.27, 48.34)	<0.0001
	14 days after the first dose of vaccine (PPS2)	329	99.70 (98.32, 99.99)	329	99.70 (98.32, 99.99)	333	99.70 (98.34, 99.99)	331	99.70 (98.33, 99.99)	0.00 (−1.42, 1.42)	1.0000	0.00 (−1.40, 1.42)	1.0000	0.00 (−1.40, 1.43)	1.0000
	14 days after the full course of immunization (PPS3)	659	99.70 (98.91, 99.96)	643	99.38 (98.41, 99.83)	653	99.54 (98.66, 99.91)	663	99.55 (98.68, 99.91)	0.32 (−0.55, 1.32)	0.4466	−0.01 (−0.94, 0.91)	1.0000	0.16 (−0.79, 1.18)	0.7239
Pre-immunization antibody-positive population	7 days after the first dose of vaccine (PPS1)	22	50.00 (28.22, 71.78)	29	34.48 (17.94, 54.33)	29	51.72 (32.53, 70.55)	22	68.18 (45.13, 86.14)	15.52 (−11.73, 41.06)	0.2648	−16.46 (−41.10, 11.09)	0.2369	1.72 (−25.27, 28.52)	0.9029
	14 days after the first dose of vaccine (PPS2)	36	97.22 (85.47, 99.93)	32	96.88 (83.78, 99.92)	39	97.44 (86.52, 99.94)	44	97.73 (87.98, 99.94)	0.35 (−11.58, 13.44)	1.0000	−0.29 (−11.26, 9.65)	1.0000	0.21 (−10.86, 12.02)	1.0000
	14 days after the full course of immunization (PPS3)	56	96.43 (87.69, 99.56)	62	93.55 (84.30, 98.21)	70	95.71 (87.98, 99.11)	66	95.45 (87.29, 99.05)	2.88 (−6.52, 12.47)	0.6818	0.26 (−7.99, 8.85)	1.0000	−0.71 (−8.88, 8.37)	1.0000
10–18 years	7 days after the first dose of vaccine (PPS1)	19	89.47 (66.86, 98.70)	14	28.57 (8.39, 58.10)	18	94.44 (72.71, 99.86)	18	94.44 (72.71, 99.86)	60.90 (28.40, 81.57)	0.0003	0.00 (−21.53, 21.53)	1.0000	4.97 (−17.34, 27.29)	1.0000
	14 days after the first dose of vaccine (PPS2)	25	100.00 (86.28, 100.00)	37	100.00 (90.51, 100.00)	31	100.00 (88.78, 100.00)	26	100.00 (86.77, 100.00)	0.00 (−13.51, 9.55)	1.0000	0.00 (−11.20, 13.07)	1.0000	0.00 (−11.20, 13.53)	1.0000
	14 days after the full course of immunization (PPS3)	44	100.00 (91.96, 100.00)	51	100.00 (93.02, 100.00)	50	100.00 (92.89, 100.00)	45	100.00 (92.13, 100.00)	0.00 (−8.11, 7.07)	1.0000	0.00 (−7.21, 7.94)	1.0000	0.00 (−7.21, 8.11)	1.0000
≥18 years	7 days after the first dose of vaccine (PPS1)	305	51.48 (45.71, 57.21)	302	32.78 (27.51, 38.39)	303	72.94 (67.56, 77.86)	305	46.56 (40.85, 52.33)	18.69 (10.88, 26.28)	<0.0001	26.38 (18.73, 33.72)	<0.0001	21.46 (13.84, 28.85)	<0.0001
	14 days after the first dose of vaccine (PPS2)	304	99.67 (98.18, 99.99)	292	99.66 (98.11, 99.99)	302	99.67 (98.17, 99.99)	305	99.67 (98.19, 99.99)	0.01 (−1.53, 1.61)	1.0000	−0.00 (−1.55, 1.53)	1.0000	−0.00 (−1.55, 1.54)	1.0000
	14 days after the full course of immunization (PPS3)	615	99.67 (98.83, 99.96)	592	99.32 (98.28, 99.82)	603	99.50 (98.55, 99.90)	618	99.51 (98.59, 99.90)	0.35 (−0.58, 1.44)	0.4436	−0.01 (−1.02, 0.98)	1.0000	−0.17 (−1.16, 0.73)	0.6840

Note: Positive conversion (4-fold increase) rate: the percentage of participants with RVNA titer that increased at least 4 times after immunization among the total number of participants with RVNA ≥ 0.5 IU/mL before immunization.

**Table A5:** Comparison of neutralizing antibody GMC levels in different the subgroup populations.

Population	Blood Sampling Time	Analysis Set	T5		C5		T4		C4		Between T5s		Between T4s		Between T4 and T5s	
			N	GMC (95% CI)	N	GMC (95% CI)	N	GMC (95% CI)	N	GMC (95% CI)	Ratio (95% CI)	P	Ratio (95% CI)	P	Ratio (95% CI)	P
Total population	7 days after the first dose of vaccine	PPS1	324	0.48 (0.41,0.55)	316	0.30 (0.26, 0.35)	321	0.85 (0.73, 0.99)	323	0.43 (0.37, 0.50)	1.58 (1.29, 1.94)	<0.0001	1.97 (1.58, 2.47)	<0.0001	1.78 (1.43, 2.20)	<0.0001
	14 days after the first dose of vaccine	PPS2	329	33.04 (30.04, 36.34)	329	30.86 (28.13, 33.86)	333	32.67 (29.71, 35.92)	331	30.99 (28.21, 34.03)	1.07 (0.94, 1.22)	0.3142	1.05 (0.92, 1.20)	0.4364	0.99 (0.86, 1.13)	0.8691
	14 days after the full course of immunization	PPS3	659	14.45 (13.56, 15.41)	643	15.51 (14.54, 16.54)	653	15.37 (14.34, 16.48)	663	15.42 (14.44, 16.46)	0.93 (0.85, 1.02)	0.1263	1.00 (0.91, 1.10)	0.9514	1.06 (0.97, 1.17)	0.1982
Pre-immunization antibody-positive population	7 days after the first dose of vaccine	PPS1	22	4.99 (2.44, 10.21)	29	2.42 (1.52, 3.85)	29	7.48 (4.04, 13.85)	22	7.76 (3.59, 16.78)	2.07 (0.93, 4.59)	0.0739	0.96 (0.37, 2.49)	0.9383	1.50 (0.60, 3.75)	0.3801
	14 days after the first dose of vaccine	PPS2	36	39.69 (28.48, 55.31)	32	34.77 (25.24, 47.89)	39	50.36 (34.30, 73.94)	44	41.49 (30.70, 56.07)	1.14 (0.72, 1.80)	0.5635	1.21 (0.75, 1.95)	0.4197	1.27 (0.77, 2.10)	0.3485
	14 days after the full course of immunization	PPS3	56	20.84 (15.96, 27.22)	62	21.45 (17.22, 26.71)	70	24.54 (18.66, 32.26)	66	27.16 (20.17, 36.58)	0.97 (0.69, 1.36)	0.8678	0.90 (0.61, 1.35)	0.6164	1.18 (0.80, 1.73)	0.4022
10–18 years	7 days after the first dose of vaccine	PPS1	19	1.17 (0.62, 2.21)	14	0.37 (0.21, 0.63)	18	1.33 (0.82, 2.17)	18	1.02 (0.51, 2.04)	3.20 (1.38, 7.40)	0.0083	1.31 (0.58, 2.97)	0.5076	1.14 (0.52, 2.48)	0.7398
	14 days after the first dose of vaccine	PPS2	25	44.10 (30.62, 63.51)	37	63.07 (48.08, 82.73)	31	53.75 (39.57, 73.02)	26	53.35 (39.48, 72.11)	0.70 (0.45, 1.08)	0.1061	1.01 (0.66, 1.54)	0.9719	1.22 (0.77, 1.93)	0.3940
	14 days after the full course of immunization	PPS3	44	23.33 (18.49, 29.44)	51	24.28 (20.99, 28.08)	50	26.99 (21.64, 33.65)	45	24.33 (19.06, 31.06)	0.96 (0.74, 1.25)	0.7637	1.11 (0.80, 1.53)	0.5275	1.16 (0.84, 1.59)	0.3631
≥18 years	7 days after the first dose of vaccine	PPS1	305	0.45 (0.39, 0.52)	302	0.30 (0.26, 0.35)	303	0.83 (0.70, 0.97)	305	0.41 (0.35, 0.48)	1.51 (1.23, 1.86)	0.0001	2.02 (1.61, 2.55)	<0.0001	1.83 (1.47, 2.28)	<0.0001
	14 days after the first dose of vaccine	PPS2	304	32.26 (29.23, 35.61)	292	28.19 (25.66, 30.97)	302	31.04 (28.12, 34.26)	305	29.58 (26.84, 32.60)	1.14 (1.00, 1.31)	0.0526	1.05 (0.91, 1.20)	0.4949	0.96 (0.84, 1.11)	0.5862
	14 days after the full course of immunization	PPS3	615	13.97 (13.08, 14.91)	592	14.92 (13.94, 15.97)	603	14.67 (13.66, 15.77)	618	14.92 (13.95, 15.96)	0.94 (0.85, 1.03)	0.1685	0.98 (0.89, 1.09)	0.7436	1.05 (0.95, 1.16)	0.3186
